# 5-Chloro-2-hy­droxy­benzaldehyde thio­semicarbazone

**DOI:** 10.1107/S1600536810043448

**Published:** 2010-10-30

**Authors:** Hadi Kargar, Reza Kia, Mehmet Akkurt, Orhan Büyükgüngör

**Affiliations:** a Department of Chemistry, School of Science, Payame Noor University (PNU), Ardakan, Yazd, Iran; b Department of Chemistry, Science and Research Branch, Islamic Azad University, Tehran, Iran; c Department of Physics, Faculty of Arts and Sciences, Erciyes University, 38039 Kayseri, Turkey; d Department of Physics, Faculty of Arts and Sciences, Ondokuz Mayıs University, 55139 Samsun, Turkey

## Abstract

In the title compound, C_8_H_8_ClN_3_OS, the whole mol­ecule assumes a planar structure, with an r.m.s. deviation of 0.108 (2) Å, and an intra­molecular O—H⋯N hydrogen bond generates and *S*(6) and ring motif. In the crystal structure, each of two pairs of inter­molecular N—H⋯S hydrogen bonds connects two mol­ecules, forming inversion dimers with *R*_2_^2^(8) motifs.

## Related literature

For the biological activities and pharmaceutical properties of thio­semicarbazones and their derivatives, see: Casas *et al.* (2000 ▶); Ferrari *et al.* (2000 ▶); Maccioni *et al.* (2003 ▶). For bond-length data, see: Allen *et al.* (1987 ▶). For hydrogen-bond motifs, see: Bernstein *et al.* (1995 ▶).
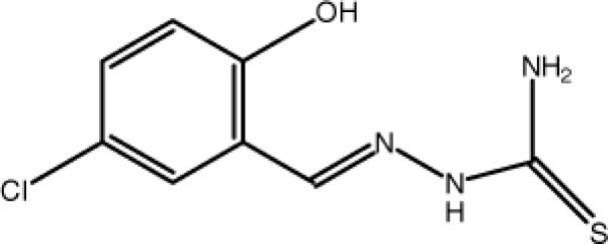


## Experimental

### 

#### Crystal data

C_8_H_8_ClN_3_OS
*M* *_r_* = 229.69
Monoclinic, 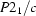

*a* = 5.8303 (4) Å
*b* = 23.6579 (17) Å
*c* = 7.5893 (5) Å
β = 104.164 (6)°
*V* = 1014.99 (12) Å^3^
*Z* = 4
Mo *K*α radiation
μ = 0.55 mm^−1^
*T* = 296 K
0.52 × 0.33 × 0.08 mm

#### Data collection

Stoe IPDS II diffractometer
Absorption correction: integration (*X-RED32*; Stoe & Cie, 2002 ▶) *T*_min_ = 0.763, *T*_max_ = 0.957
4469 measured reflections
1895 independent reflections
1524 reflections with *I* > 2σ(*I*)
*R*_int_ = 0.051

#### Refinement

*R*[*F* ^2^ > 2σ(*F* ^2^)] = 0.044
*wR*(*F* ^2^) = 0.120
*S* = 1.04
1895 reflections
128 parameters
H-atom parameters constrained
Δρ_max_ = 0.20 e Å^−3^
Δρ_min_ = −0.37 e Å^−3^

### 

Data collection: *X-AREA* (Stoe & Cie, 2002 ▶); cell refinement: *X-AREA*; data reduction: *X-RED32* (Stoe & Cie, 2002 ▶); program(s) used to solve structure: *SIR97* (Altomare *et al.*, 1999 ▶); program(s) used to refine structure: *SHELXL97* (Sheldrick, 2008 ▶); molecular graphics: *ORTEP-3* (Farrugia, 1997 ▶); software used to prepare material for publication: *WinGX* (Farrugia, 1999 ▶).

## Supplementary Material

Crystal structure: contains datablocks global, I. DOI: 10.1107/S1600536810043448/is2621sup1.cif

Structure factors: contains datablocks I. DOI: 10.1107/S1600536810043448/is2621Isup2.hkl

Additional supplementary materials:  crystallographic information; 3D view; checkCIF report

## Figures and Tables

**Table 1:** Hydrogen-bond geometry (Å, °)

*D*—H⋯*A*	*D*—H	H⋯*A*	*D*⋯*A*	*D*—H⋯*A*
N2—H2⋯S1^i^	0.86	2.70	3.491 (2)	153
N3—H3*A*⋯S1^ii^	0.86	2.87	3.387 (2)	120
N3—H3*A*⋯N1	0.86	2.36	2.693 (3)	103
N3—H3*B*⋯S1^iii^	0.86	2.55	3.390 (2)	167

Symmetry codes: (i) 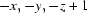; (ii) 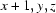; (iii) 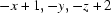.

**Fig. 1.**
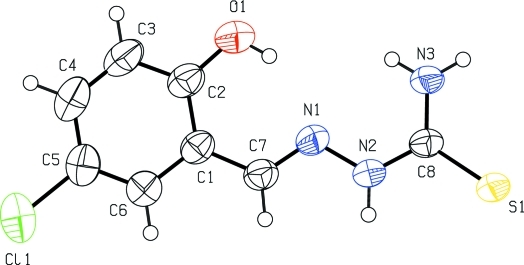
The title molecule with the atom numbering scheme. Displacement ellipsoids for non-H atoms are drawn at the 50% probability level.

**Fig. 2.**
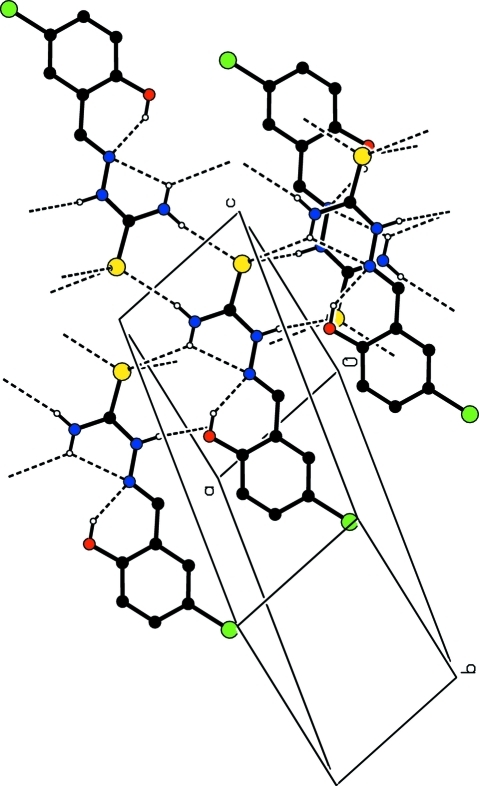
View of the packing and hydrogen bonding interactions of (I), showing dimer formation by R22(8) ring motif. All H atoms not involved in hydrogen bonding have been omitted for clarity.

## supplementary crystallographic information

### Comment

Thiosemicarbazones constitute an important class of *N*,*S* donor ligands due to their propensity to react with a wide range of metals (Casas *et al.*, 2000). Thiosemicarbazones exhibit various biological activities and have therefore attracted considerable pharmaceutical interest (Maccioni *et al.*, 2003; Ferrari *et al.*, 2000). We here report the crystal structure of the title compound (I).

The title molecule (I) shown in Fig. 1 is planar with an r.m.s. deviation of 0.108 Å and all bond lengths agree with standard values (Allen *et al.*, 1987). Intramolecular O—H···N and N—H···N hydrogen bonds (Table 1) generate the *S*(6) and *S*(5) ring motifs, respectively (Bernstein *et al.*, 1995).

In the crystal structure, molecules are linked by N—H···S hydrogen bonds, forming *R*_2_^2^(8) dimers (Table 1 and Fig. 2).

### Experimental

A mixture of 5-chlorosalicylalehyde (0.01 mol) and hydrazinecarbothioamide (0.01 mol) in 20 ml of ethanol was refluxed for about 2 h. On cooling, the solid separated was filtered and recrystallized from ethanol. Crystals of (I) suitable for X-ray diffraction were obtained by slow evaporation of ethanol.

### Refinement

All H atoms were geometrically placed (C—H = 0.93 Å, N—H = 0.86 Å and O—H = 0.82 Å) and refined as riding with *U*_iso_(H) = 1.2*U*_eq_(C,N) and 1.5*U*_eq_(O).

### Crystal data

**Table:**

C_8_H_8_ClN_3_OS	*F*(000) = 472
*M**_r_* = 229.69	*D*_x_ = 1.503 Mg m^−^^3^
Monoclinic, *P*2_1_/*c*	Mo *K*α radiation, λ = 0.71073 Å
Hall symbol: -P 2ybc	Cell parameters from 8344 reflections
*a* = 5.8303 (4) Å	θ = 1.7–26.2°
*b* = 23.6579 (17) Å	µ = 0.55 mm^−^^1^
*c* = 7.5893 (5) Å	*T* = 296 K
β = 104.164 (6)°	Prism, colourless
*V* = 1014.99 (12) Å^3^	0.52 × 0.33 × 0.08 mm
*Z* = 4	

### Data collection

**Table:**

Stoe IPDS II diffractometer	1895 independent reflections
Radiation source: sealed X-ray tube, 12 x 0.4 mm long-fine focus	1524 reflections with *I* > 2σ(*I*)
plane graphite	*R*_int_ = 0.051
Detector resolution: 6.67 pixels mm^-1^	θ_max_ = 25.6°, θ_min_ = 1.7°
ω scans	*h* = −5→7
Absorption correction: integration (*X-RED32*; Stoe & Cie, 2002)	*k* = −28→26
*T*_min_ = 0.763, *T*_max_ = 0.957	*l* = −9→9
4469 measured reflections	

### Refinement

**Table:**

Refinement on *F*^2^	Primary atom site location: structure-invariant direct methods
Least-squares matrix: full	Secondary atom site location: difference Fourier map
*R*[*F*^2^ > 2σ(*F*^2^)] = 0.044	Hydrogen site location: inferred from neighbouring sites
*wR*(*F*^2^) = 0.120	H-atom parameters constrained
*S* = 1.04	*w* = 1/[σ^2^(*F*_o_^2^) + (0.0681*P*)^2^ + 0.120*P*] where *P* = (*F*_o_^2^ + 2*F*_c_^2^)/3
1895 reflections	(Δ/σ)_max_ < 0.001
128 parameters	Δρ_max_ = 0.20 e Å^−^^3^
0 restraints	Δρ_min_ = −0.36 e Å^−^^3^

### Special details

**Table:**

Geometry. Bond distances, angles *etc*. have been calculated using the rounded fractional coordinates. All su's are estimated from the variances of the (full) variance-covariance matrix. The cell e.s.d.'s are taken into account in the estimation of distances, angles and torsion angles
Refinement. Refinement on *F*^2^ for ALL reflections except those flagged by the user for potential systematic errors. Weighted *R*-factors *wR* and all goodnesses of fit *S* are based on *F*^2^, conventional *R*-factors *R* are based on *F*, with *F* set to zero for negative *F*^2^. The observed criterion of *F*^2^ > σ(*F*^2^) is used only for calculating -*R*-factor-obs *etc*. and is not relevant to the choice of reflections for refinement. *R*-factors based on *F*^2^ are statistically about twice as large as those based on *F*, and *R*-factors based on ALL data will be even larger.

### Fractional atomic coordinates and isotropic or equivalent isotropic displacement parameters (Å^2^)

**Table:**

	*x*	*y*	*z*	*U*_iso_*/*U*_eq_	
Cl1	0.36123 (17)	0.20555 (4)	−0.30970 (10)	0.0868 (3)	
S1	0.15507 (9)	0.01021 (3)	0.79551 (8)	0.0559 (2)	
O1	0.8537 (3)	0.13549 (11)	0.4272 (3)	0.0750 (8)	
N1	0.4525 (3)	0.08332 (9)	0.4467 (3)	0.0489 (6)	
N2	0.3002 (3)	0.05468 (10)	0.5260 (3)	0.0523 (6)	
N3	0.5874 (3)	0.04298 (10)	0.7883 (3)	0.0524 (7)	
C1	0.4996 (4)	0.13306 (11)	0.1830 (3)	0.0493 (8)	
C2	0.7333 (4)	0.15067 (12)	0.2573 (4)	0.0573 (8)	
C3	0.8480 (5)	0.18395 (14)	0.1559 (4)	0.0687 (10)	
C4	0.7368 (6)	0.20095 (13)	−0.0164 (4)	0.0702 (10)	
C5	0.5049 (5)	0.18372 (12)	−0.0912 (4)	0.0604 (9)	
C6	0.3886 (5)	0.15099 (11)	0.0062 (3)	0.0542 (8)	
C7	0.3665 (4)	0.09979 (11)	0.2818 (3)	0.0507 (8)	
C8	0.3636 (4)	0.03756 (11)	0.6999 (3)	0.0454 (7)	
H1	0.76270	0.12040	0.48000	0.1120*	
H2	0.15960	0.04740	0.46260	0.0630*	
H3	1.00350	0.19500	0.20550	0.0830*	
H3A	0.68790	0.05710	0.73440	0.0630*	
H3B	0.63280	0.03240	0.89980	0.0630*	
H4	0.81520	0.22370	−0.08260	0.0840*	
H6	0.23310	0.14030	−0.04510	0.0650*	
H7	0.21210	0.08970	0.22430	0.0610*	

### Atomic displacement parameters (Å^2^)

**Table:**

	*U*^11^	*U*^22^	*U*^33^	*U*^12^	*U*^13^	*U*^23^
Cl1	0.1118 (7)	0.0820 (6)	0.0700 (4)	0.0054 (5)	0.0287 (4)	0.0188 (4)
S1	0.0349 (3)	0.0795 (5)	0.0526 (3)	−0.0048 (3)	0.0096 (2)	0.0063 (3)
O1	0.0458 (10)	0.0951 (17)	0.0791 (12)	−0.0077 (10)	0.0058 (9)	0.0132 (11)
N1	0.0379 (9)	0.0554 (12)	0.0544 (10)	−0.0011 (8)	0.0131 (8)	0.0019 (9)
N2	0.0336 (9)	0.0683 (14)	0.0529 (10)	−0.0059 (9)	0.0065 (8)	0.0074 (9)
N3	0.0334 (9)	0.0742 (15)	0.0485 (10)	−0.0031 (9)	0.0079 (7)	0.0017 (9)
C1	0.0447 (12)	0.0466 (14)	0.0597 (13)	0.0017 (10)	0.0189 (10)	−0.0007 (10)
C2	0.0453 (12)	0.0558 (16)	0.0729 (15)	0.0009 (11)	0.0186 (11)	0.0020 (12)
C3	0.0489 (14)	0.0623 (18)	0.100 (2)	−0.0044 (12)	0.0281 (14)	0.0079 (15)
C4	0.0725 (17)	0.0577 (18)	0.0926 (19)	0.0020 (14)	0.0438 (15)	0.0108 (15)
C5	0.0729 (17)	0.0490 (15)	0.0653 (14)	0.0056 (13)	0.0283 (12)	0.0030 (11)
C6	0.0540 (13)	0.0505 (15)	0.0595 (12)	0.0029 (11)	0.0168 (10)	−0.0014 (11)
C7	0.0403 (12)	0.0544 (15)	0.0570 (12)	0.0002 (10)	0.0111 (10)	0.0015 (11)
C8	0.0344 (10)	0.0510 (14)	0.0505 (11)	0.0009 (9)	0.0096 (9)	−0.0029 (10)

### Geometric parameters (Å, °)

**Table:**

Cl1—C5	1.743 (3)	C1—C2	1.404 (4)
S1—C8	1.690 (2)	C1—C6	1.405 (3)
O1—C2	1.357 (4)	C1—C7	1.438 (3)
O1—H1	0.8200	C2—C3	1.383 (4)
N1—N2	1.368 (3)	C3—C4	1.370 (4)
N1—C7	1.289 (3)	C4—C5	1.393 (5)
N2—C8	1.343 (3)	C5—C6	1.361 (4)
N3—C8	1.319 (3)	C3—H3	0.9300
N2—H2	0.8600	C4—H4	0.9300
N3—H3A	0.8600	C6—H6	0.9300
N3—H3B	0.8600	C7—H7	0.9300
			
Cl1···C5^i^	3.606 (3)	C7···C8^vi^	3.598 (4)
S1···N3^ii^	3.387 (2)	C7···N3^vi^	3.440 (4)
S1···N2^iii^	3.491 (2)	C7···C3^ii^	3.550 (4)
S1···N3^iv^	3.390 (2)	C8···C7^vi^	3.598 (4)
S1···H3A^ii^	2.8700	C8···C6^vii^	3.530 (3)
S1···H2^iii^	2.7000	C8···N1^vi^	3.339 (3)
S1···H3B^iv^	2.5500	C3···H7^v^	3.0300
S1···H7^iii^	3.1700	C3···H4^viii^	2.9900
O1···N1	2.681 (3)	C7···H3^ii^	3.0500
O1···N2^v^	3.168 (3)	C7···H1	2.4800
O1···H2^v^	2.7100	H1···N1	1.9700
N1···O1	2.681 (3)	H1···C7	2.4800
N1···N3	2.693 (3)	H1···H3A	2.5600
N1···C8^vi^	3.339 (3)	H2···O1^ii^	2.7100
N2···O1^ii^	3.168 (3)	H2···H7	2.1500
N2···S1^iii^	3.491 (2)	H2···S1^iii^	2.7000
N3···C6^vii^	3.398 (3)	H3···C7^v^	3.0500
N3···S1^iv^	3.390 (2)	H3A···S1^v^	2.8700
N3···S1^v^	3.387 (2)	H3A···N1	2.3600
N3···N1	2.693 (3)	H3A···H1	2.5600
N3···C7^vi^	3.440 (4)	H3B···S1^iv^	2.5500
N1···H1	1.9700	H4···C3^i^	2.9900
N1···H3A	2.3600	H6···H7	2.4000
C3···C7^v^	3.550 (4)	H7···C3^ii^	3.0300
C5···Cl1^viii^	3.606 (3)	H7···H2	2.1500
C6···N3^ix^	3.398 (3)	H7···H6	2.4000
C6···C8^ix^	3.530 (3)	H7···S1^iii^	3.1700
			
C2—O1—H1	109.00	Cl1—C5—C4	119.6 (2)
N2—N1—C7	115.9 (2)	Cl1—C5—C6	119.9 (2)
N1—N2—C8	122.0 (2)	C4—C5—C6	120.5 (3)
C8—N2—H2	119.00	C1—C6—C5	121.1 (3)
N1—N2—H2	119.00	N1—C7—C1	122.7 (2)
H3A—N3—H3B	120.00	N2—C8—N3	118.1 (2)
C8—N3—H3A	120.00	S1—C8—N2	118.92 (18)
C8—N3—H3B	120.00	S1—C8—N3	123.01 (18)
C2—C1—C6	118.0 (2)	C2—C3—H3	119.00
C6—C1—C7	118.8 (2)	C4—C3—H3	119.00
C2—C1—C7	123.2 (2)	C3—C4—H4	120.00
O1—C2—C1	121.9 (2)	C5—C4—H4	120.00
C1—C2—C3	120.0 (3)	C1—C6—H6	119.00
O1—C2—C3	118.1 (2)	C5—C6—H6	119.00
C2—C3—C4	121.2 (3)	N1—C7—H7	119.00
C3—C4—C5	119.2 (3)	C1—C7—H7	119.00
			
C7—N1—N2—C8	−176.7 (2)	C6—C1—C2—C3	−0.9 (4)
N2—N1—C7—C1	176.5 (2)	C7—C1—C2—O1	2.5 (4)
N1—N2—C8—S1	171.99 (18)	O1—C2—C3—C4	−179.8 (3)
N1—N2—C8—N3	−8.0 (4)	C1—C2—C3—C4	0.9 (5)
C7—C1—C2—C3	−178.2 (3)	C2—C3—C4—C5	−0.8 (5)
C2—C1—C6—C5	1.0 (4)	C3—C4—C5—Cl1	179.5 (2)
C7—C1—C6—C5	178.3 (3)	C3—C4—C5—C6	0.8 (5)
C2—C1—C7—N1	−0.3 (4)	Cl1—C5—C6—C1	−179.6 (2)
C6—C1—C7—N1	−177.6 (2)	C4—C5—C6—C1	−0.9 (4)
C6—C1—C2—O1	179.8 (2)		

Symmetry codes: (i) *x*, −*y*+1/2, *z*−1/2; (ii) *x*−1, *y*, *z*; (iii) −*x*, −*y*, −*z*+1; (iv) −*x*+1, −*y*, −*z*+2; (v) *x*+1, *y*, *z*; (vi) −*x*+1, −*y*, −*z*+1; (vii) *x*, *y*, *z*+1; (viii) *x*, −*y*+1/2, *z*+1/2; (ix) *x*, *y*, *z*−1.

### Hydrogen-bond geometry (Å, °)

**Table:**

*D*—H···*A*	*D*—H	H···*A*	*D*···*A*	*D*—H···*A*
O1—H1···N1	0.82	1.97	2.681 (3)	144
N2—H2···S1^iii^	0.86	2.70	3.491 (2)	153
N3—H3A···S1^v^	0.86	2.87	3.387 (2)	120
N3—H3A···N1	0.86	2.36	2.693 (3)	103
N3—H3B···S1^iv^	0.86	2.55	3.390 (2)	167

Symmetry codes: (iii) −*x*, −*y*, −*z*+1; (v) *x*+1, *y*, *z*; (iv) −*x*+1, −*y*, −*z*+2.
